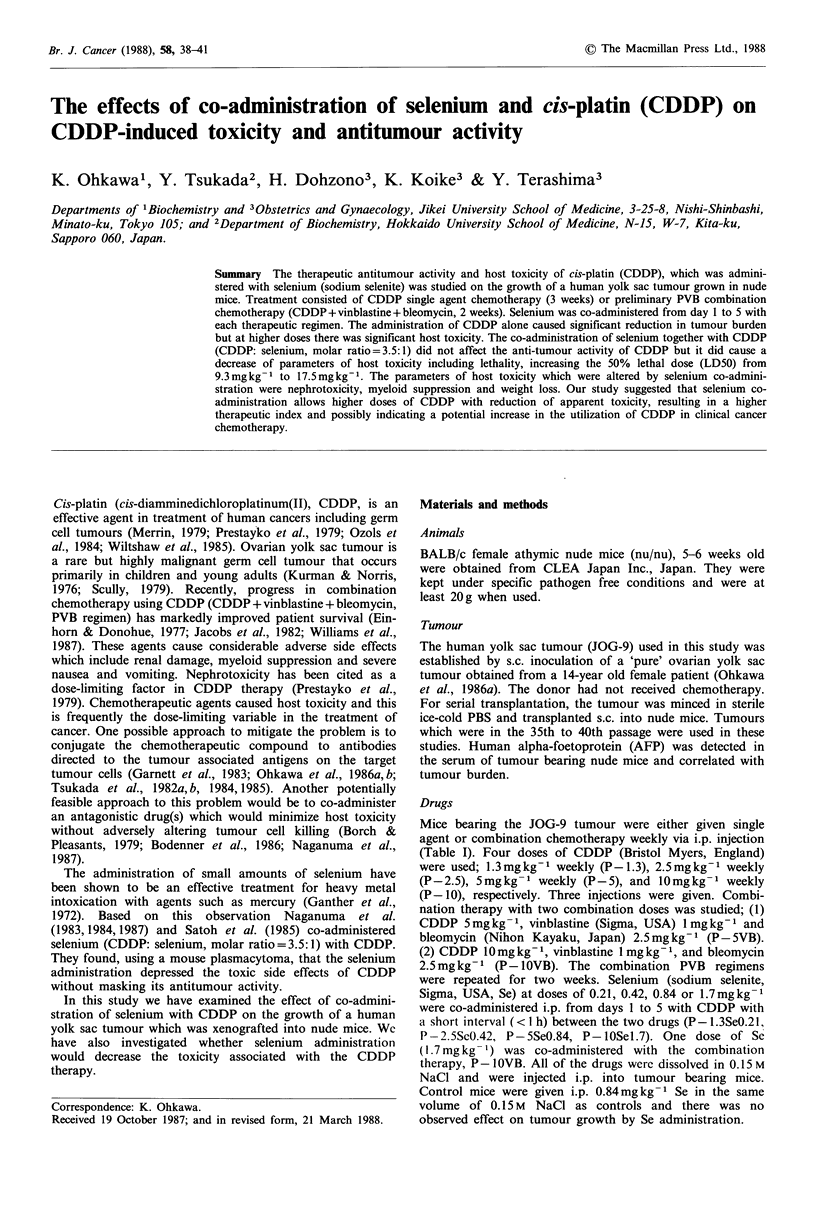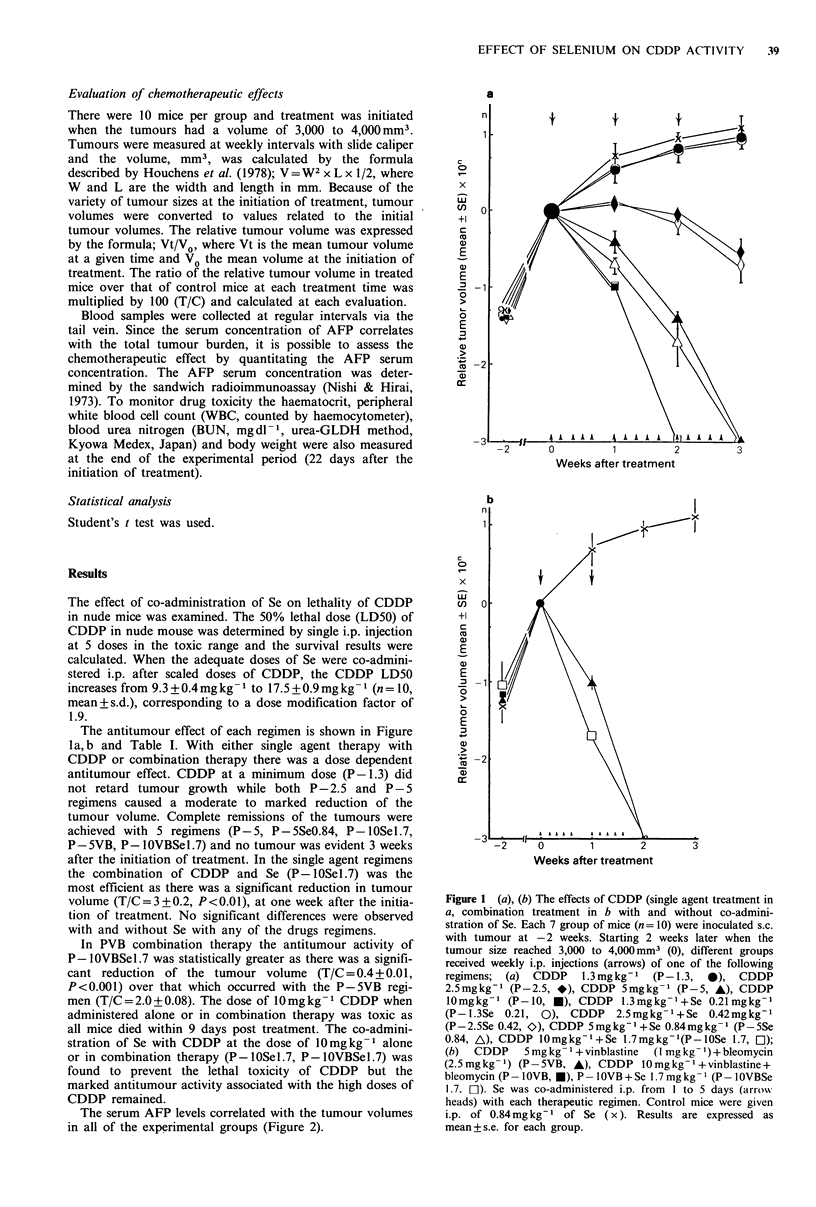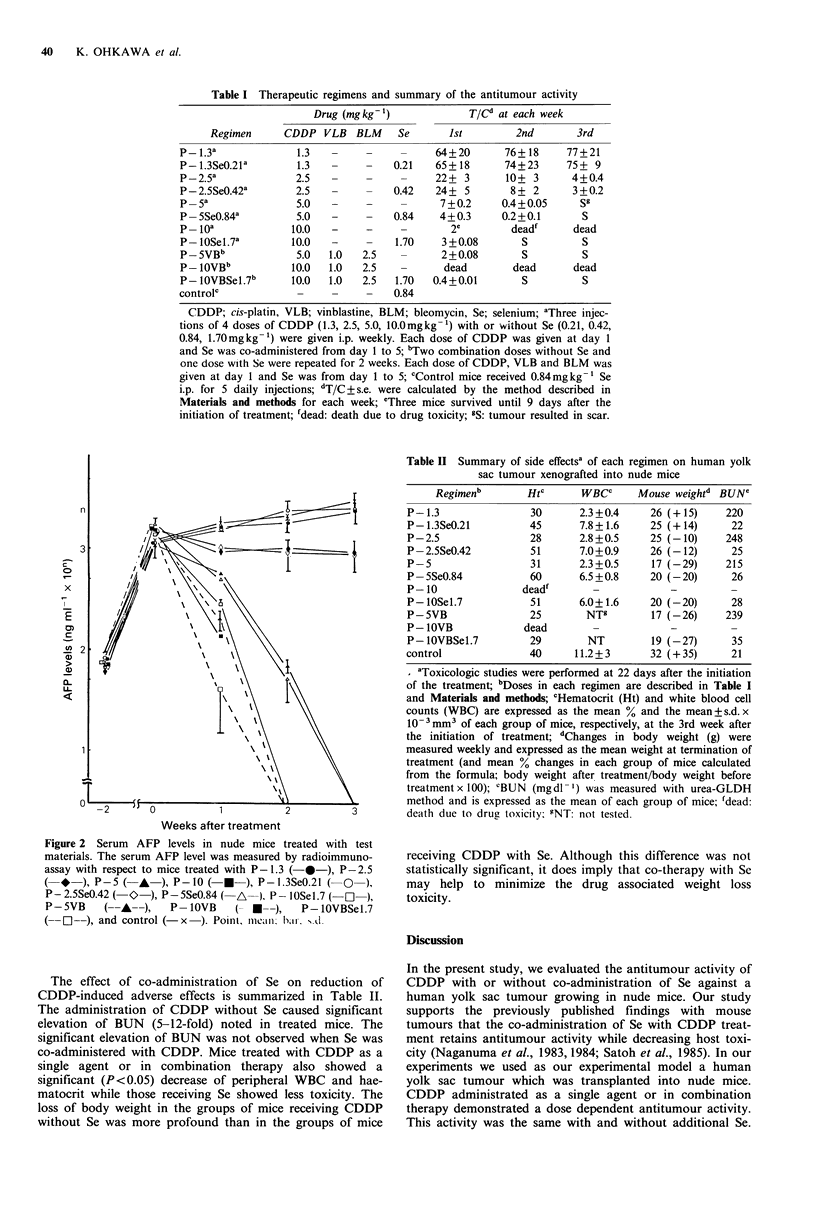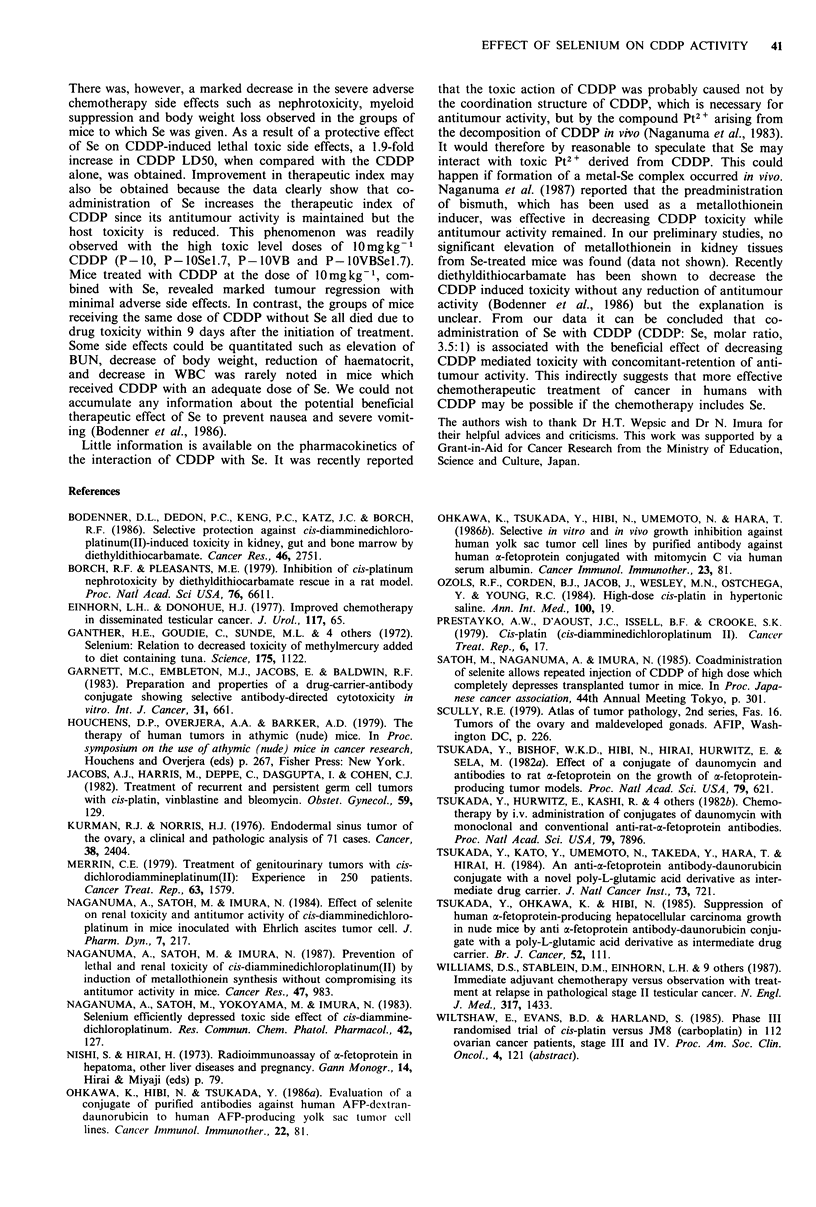# The effects of co-administration of selenium and cis-platin (CDDP) on CDDP-induced toxicity and antitumour activity.

**DOI:** 10.1038/bjc.1988.157

**Published:** 1988-07

**Authors:** K. Ohkawa, Y. Tsukada, H. Dohzono, K. Koike, Y. Terashima

**Affiliations:** Department of Biochemistry, Jikei University School of Medicine, Tokyo, Japan.

## Abstract

The therapeutic antitumour activity and host toxicity of cis-platin (CDDP), which was administered with selenium (sodium selenite) was studied on the growth of a human yolk sac tumour grown in nude mice. Treatment consisted of CDDP single agent chemotherapy (3 weeks) or preliminary PVB combination chemotherapy (CDDP + vinblastine + bleomycin, 2 weeks). Selenium was co-administered from day 1 to 5 with each therapeutic regimen. The administration of CDDP alone caused significant reduction in tumour burden but at higher doses there was significant host toxicity. The co-administration of selenium together with CDDP (CDDP: selenium, molar ratio = 3.5:1) did not affect the anti-tumour activity of CDDP but it did cause a decrease of parameters of host toxicity including lethality, increasing the 50% lethal dose (LD50) from 9.3 mg kg-1 to 17.5 mg kg-1. The parameters of host toxicity which were altered by selenium co-administration were nephrotoxicity, myeloid suppression and weight loss. Our study suggested that selenium co-administration allows higher doses of CDDP with reduction of apparent toxicity, resulting in a higher therapeutic index and possibly indicating a potential increase in the utilization of CDDP in clinical cancer chemotherapy.


*Br. J. Cancer* (1988), **58**, 38–41 © The Macmillan Press Ltd., 1988

### The effects of co-administration of selenium and *cis*-platin (CDDP) on CDDP-induced toxicity and antitumour activity

K. Ohkawa[1], Y. Tsukada[2], H. Dohzono[3], K. Koike[3] & Y. Terashima[3]

*Departments of [1]Biochemistry and [3]Obstetrics and Gynaecology, Jikei University School of Medicine, 3-25-8, Nishi-Shinbashi, Minato-ku, Tokyo 105; and [2]Department of Biochemistry, Hokkaido University School of Medicine, N-15, W-7, Kita-ku, Sapporo 060, Japan.*

**Summary** The therapeutic antitumour activity and host toxicity of *cis*-platin (CDDP), which was administered with selenium (sodium selenite) was studied on the growth of a human yolk sac tumour grown in nude mice. Treatment consisted of CDDP single agent chemotherapy (3 weeks) or preliminary PVB combination chemotherapy (CDDP + vinblastine + bleomycin, 2 weeks). Selenium was co-administered from day 1 to 5 with each therapeutic regimen. The administration of CDDP alone caused significant reduction in tumour burden but at higher doses there was significant host toxicity. The co-administration of selenium together with CDDP (CDDP: selenium, molar ratio = 3.5:1) did not affect the anti-tumour activity of CDDP but it did cause a decrease of parameters of host toxicity including lethality, increasing the 50% lethal dose (LD50) from 9.3 mg kg$^{-1}$ to 17.5 mg kg$^{-1}$. The parameters of host toxicity which were altered by selenium co-administration were nephrotoxicity, myeloid suppression and weight loss. Our study suggested that selenium co-administration allows higher doses of CDDP with reduction of apparent toxicity, resulting in a higher therapeutic index and possibly indicating a potential increase in the utilization of CDDP in clinical cancer chemotherapy.

*Cis*-platin (*cis*-diamminedichloroplatinum(II), CDDP, is an effective agent in treatment of human cancers including germ cell tumours (Merrin, 1979; Prestayko *et al.*, 1979; Ozols *et al.*, 1984; Wiltshaw *et al.*, 1985). Ovarian yolk sac tumour is a rare but highly malignant germ cell tumour that occurs primarily in children and young adults (Kurman & Norris, 1976; Scully, 1979). Recently, progress in combination chemotherapy using CDDP (CDDP + vinblastine + bleomycin, PVB regimen) has markedly improved patient survival (Einhorn & Donohue, 1977; Jacobs *et al.*, 1982; Williams *et al.*, 1987). These agents cause considerable adverse side effects which include renal damage, myeloid suppression and severe nausea and vomiting. Nephrotoxicity has been cited as a dose-limiting factor in CDDP therapy (Prestayko *et al.*, 1979). Chemotherapeutic agents caused host toxicity and this is frequently the dose-limiting variable in the treatment of cancer. One possible approach to mitigate the problem is to conjugate the chemotherapeutic compound to antibodies directed to the tumour associated antigens on the target tumour cells (Garnett *et al.*, 1983; Ohkawa *et al.*, 1986*a,b*; Tsukada *et al.*, 1982*a,b*, 1984, 1985). Another potentially feasible approach to this problem would be to co-administer an antagonistic drug(s) which would minimize host toxicity without adversely altering tumour cell killing (Borch & Pleasants, 1979; Bodenner *et al.*, 1986; Naganuma *et al.*, 1987).

The administration of small amounts of selenium have been shown to be an effective treatment for heavy metal intoxication with agents such as mercury (Ganther *et al.*, 1972). Based on this observation Naganuma *et al.* (1983, 1984, 1987) and Satoh *et al.* (1985) co-administered selenium (CDDP: selenium, molar ratio = 3.5:1) with CDDP. They found, using a mouse plasmacytoma, that the selenium administration depressed the toxic side effects of CDDP without masking its antitumour activity.

In this study we have examined the effect of co-administration of selenium with CDDP on the growth of a human yolk sac tumour which was xenografted into nude mice. We have also investigated whether selenium administration would decrease the toxicity associated with the CDDP therapy.

Correspondence: K. Ohkawa.
Received 19 October 1987; and in revised form, 21 March 1988.

### Materials and methods

### *Animals*

BALB/c female athymic nude mice (nu/nu), 5–6 weeks old were obtained from CLEA Japan Inc., Japan. They were kept under specific pathogen free conditions and were at least 20 g when used.

### *Tumour*

The human yolk sac tumour (JOG-9) used in this study was established by s.c. inoculation of a 'pure' ovarian yolk sac tumour obtained from a 14-year old female patient (Ohkawa *et al.*, 1986*a*). The donor had not received chemotherapy. For serial transplantation, the tumour was minced in sterile ice-cold PBS and transplanted s.c. into nude mice. Tumours which were in the 35th to 40th passage were used in these studies. Human alpha-foetoprotein (AFP) was detected in the serum of tumour bearing nude mice and correlated with tumour burden.

### *Drugs*

Mice bearing the JOG-9 tumour were either given single agent or combination chemotherapy weekly via i.p. injection (Table I). Four doses of CDDP (Bristol Myers, England) were used; 1.3 mg kg$^{-1}$ weekly (P–1.3), 2.5 mg kg$^{-1}$ weekly (P–2.5), 5 mg kg$^{-1}$ weekly (P–5), and 10 mg kg$^{-1}$ weekly (P–10), respectively. Three injections were given. Combination therapy with two combination doses was studied; (1) CDDP 5 mg kg$^{-1}$, vinblastine (Sigma, USA) 1 mg kg$^{-1}$ and bleomycin (Nihon Kayaku, Japan) 2.5 mg kg$^{-1}$ (P–5VB). (2) CDDP 10 mg kg$^{-1}$, vinblastine 1 mg kg$^{-1}$, and bleomycin 2.5 mg kg$^{-1}$ (P–10VB). The combination PVB regimens were repeated for two weeks. Selenium (sodium selenite, Sigma, USA, Se) at doses of 0.21, 0.42, 0.84 or 1.7 mg kg$^{-1}$ were co-administered i.p. from days 1 to 5 with CDDP with a short interval (< 1 h) between the two drugs (P–1.3Se0.21, P–2.5Se0.42, P–5Se0.84, P–10Se1.7). One dose of Se (1.7 mg kg$^{-1}$) was co-administered with the combination therapy, P–10VB. All of the drugs were dissolved in 0.15 M NaCl and were injected i.p. into tumour bearing mice. Control mice were given i.p. 0.84 mg kg$^{-1}$ Se in the same volume of 0.15 M NaCl as controls and there was no observed effect on tumour growth by Se administration.

*Evaluation of chemotherapeutic effects*

There were 10 mice per group and treatment was initiated when the tumours had a volume of 3,000 to 4,000 $mm^3$. Tumours were measured at weekly intervals with slide caliper and the volume, $mm^3$, was calculated by the formula described by Houchens *et al.* (1978); $V = W^2 \times L \times 1/2$, where W and L are the width and length in mm. Because of the variety of tumour sizes at the initiation of treatment, tumour volumes were converted to values related to the initial tumour volumes. The relative tumour volume was expressed by the formula; $Vt/V_0$, where Vt is the mean tumour volume at a given time and $V_0$ the mean volume at the initiation of treatment. The ratio of the relative tumour volume in treated mice over that of control mice at each treatment time was multiplied by 100 (T/C) and calculated at each evaluation.

Blood samples were collected at regular intervals via the tail vein. Since the serum concentration of AFP correlates with the total tumour burden, it is possible to assess the chemotherapeutic effect by quantitating the AFP serum concentration. The AFP serum concentration was determined by the sandwich radioimmunoassay (Nishi & Hirai, 1973). To monitor drug toxicity the haematocrit, peripheral white blood cell count (WBC, counted by haemocytometer), blood urea nitrogen (BUN, $mg\,dl^{-1}$, urea-GLDH method, Kyowa Medex, Japan) and body weight were also measured at the end of the experimental period (22 days after the initiation of treatment).

*Statistical analysis*

Student's *t* test was used.

### Results

The effect of co-administration of Se on lethality of CDDP in nude mice was examined. The 50% lethal dose (LD50) of CDDP in nude mouse was determined by single i.p. injection at 5 doses in the toxic range and the survival results were calculated. When the adequate doses of Se were co-administered i.p. after scaled doses of CDDP, the CDDP LD50 increases from $9.3 \pm 0.4\,mg\,kg^{-1}$ to $17.5 \pm 0.9\,mg\,kg^{-1}$ ($n = 10$, mean ± s.d.), corresponding to a dose modification factor of 1.9.

The antitumour effect of each regimen is shown in Figure 1a, b and Table I. With either single agent therapy with CDDP or combination therapy there was a dose dependent antitumour effect. CDDP at a minimum dose (P−1.3) did not retard tumour growth while both P−2.5 and P−5 regimens caused a moderate to marked reduction of the tumour volume. Complete remissions of the tumours were achieved with 5 regimens (P−5, P−5Se0.84, P−10Se1.7, P−5VB, P−10VBSe1.7) and no tumour was evident 3 weeks after the initiation of treatment. In the single agent regimens the combination of CDDP and Se (P−10Se1.7) was the most efficient as there was a significant reduction in tumour volume ($T/C = 3 \pm 0.2$, $P < 0.01$), at one week after the initiation of treatment. No significant differences were observed with and without Se with any of the drugs regimens.

In PVB combination therapy the antitumour activity of P−10VBSe1.7 was statistically greater as there was a significant reduction of the tumour volume ($T/C = 0.4 \pm 0.01$, $P < 0.001$) over that which occurred with the P−5VB regimen ($T/C = 2.0 \pm 0.08$). The dose of $10\,mg\,kg^{-1}$ CDDP when administered alone or in combination therapy was toxic as all mice died within 9 days post treatment. The co-administration of Se with CDDP at the dose of $10\,mg\,kg^{-1}$ alone or in combination therapy (P−10Se1.7, P−10VBSe1.7) was found to prevent the lethal toxicity of CDDP but the marked antitumour activity associated with the high doses of CDDP remained.

The serum AFP levels correlated with the tumour volumes in all of the experimental groups (Figure 2).

**Figure 1** (*a*), (*b*) The effects of CDDP (single agent treatment in *a*, combination treatment in *b* with and without co-administration of Se. Each 7 group of mice ($n = 10$) were inoculated s.c. with tumour at −2 weeks. Starting 2 weeks later when the tumour size reached 3,000 to 4,000 $mm^3$ (0), different groups received weekly i.p. injections (arrows) of one of the following regimens; (*a*) CDDP $1.3\,mg\,kg^{-1}$ (P−1.3, ●), CDDP $2.5\,mg\,kg^{-1}$ (P−2.5, ◆), CDDP $5\,mg\,kg^{-1}$ (P−5, ▲), CDDP $10\,mg\,kg^{-1}$ (P−10, ■), CDDP $1.3\,mg\,kg^{-1}$ + Se $0.21\,mg\,kg^{-1}$ (P−1.3Se 0.21, ○), CDDP $2.5\,mg\,kg^{-1}$ + Se $0.42\,mg\,kg^{-1}$ (P−2.5Se 0.42, ◇), CDDP $5\,mg\,kg^{-1}$ + Se $0.84\,mg\,kg^{-1}$ (P−5Se 0.84, △), CDDP $10\,mg\,kg^{-1}$ + Se $1.7\,mg\,kg^{-1}$(P−10Se 1.7, □); (*b*) CDDP $5\,mg\,kg^{-1}$ + vinblastine ($1\,mg\,kg^{-1}$) + bleomycin ($2.5\,mg\,kg^{-1}$) (P−5VB, ▲), CDDP $10\,mg\,kg^{-1}$ + vinblastine + bleomycin (P−10VB, ■), P−10VB + Se $1.7\,mg\,kg^{-1}$ (P−10VBSe 1.7, □). Se was co-administered i.p. from 1 to 5 days (arrow heads) with each therapeutic regimen. Control mice were given i.p. of $0.84\,mg\,kg^{-1}$ of Se (×). Results are expressed as mean ± s.e. for each group.

**Table I** Therapeutic regimens and summary of the antitumour activity

| Regimen | *Drug* ($mg\,kg^{-1}$) | | | | $T/C^d$ *at each week* | | |
|---|---|---|---|---|---|---|---|
| | *CDDP* | *VLB* | *BLM* | *Se* | *1st* | *2nd* | *3rd* |
| P−1.3[a] | 1.3 | − | − | − | 64±20 | 76±18 | 77±21 |
| P−1.3Se0.21[a] | 1.3 | − | − | 0.21 | 65±18 | 74±23 | 75± 9 |
| P−2.5[a] | 2.5 | − | − | − | 22± 3 | 10± 3 | 4±0.4 |
| P−2.5Se0.42[a] | 2.5 | − | − | 0.42 | 24± 5 | 8± 2 | 3±0.2 |
| P−5[a] | 5.0 | − | − | − | 7±0.2 | 0.4±0.05 | S[g] |
| P−5Se0.84[a] | 5.0 | − | − | 0.84 | 4±0.3 | 0.2±0.1 | S |
| P−10[a] | 10.0 | − | − | − | 2[e] | dead[f] | dead |
| P−10Se1.7[a] | 10.0 | − | − | 1.70 | 3±0.08 | S | S |
| P−5VB[b] | 5.0 | 1.0 | 2.5 | − | 2±0.08 | S | S |
| P−10VB[b] | 10.0 | 1.0 | 2.5 | − | dead | dead | dead |
| P−10VBSe1.7[b] | 10.0 | 1.0 | 2.5 | 1.70 | 0.4±0.01 | S | S |
| control[c] | − | − | − | 0.84 | | | |

CDDP; *cis*-platin, VLB; vinblastine, BLM; bleomycin, Se; selenium; [a]Three injections of 4 doses of CDDP (1.3, 2.5, 5.0, 10.0 $mg\,kg^{-1}$) with or without Se (0.21, 0.42, 0.84, 1.70 $mg\,kg^{-1}$) were given i.p. weekly. Each dose of CDDP was given at day 1 and Se was co-administered from day 1 to 5; [b]Two combination doses without Se and one dose with Se were repeated for 2 weeks. Each dose of CDDP, VLB and BLM was given at day 1 and Se was from day 1 to 5; [c]Control mice received 0.84 $mg\,kg^{-1}$ Se i.p. for 5 daily injections; [d]T/C±s.e. were calculated by the method described in **Materials and methods** for each week; [e]Three mice survived until 9 days after the initiation of treatment; [f]dead: death due to drug toxicity; [g]S: tumour resulted in scar.

**Figure 2** Serum AFP levels in nude mice treated with test materials. The serum AFP level was measured by radioimmunoassay with respect to mice treated with P−1.3 (—●—), P−2.5 (—◆—), P−5 (—▲—), P−10 (—■—), P−1.3Se0.21 (—○—), P−2.5Se0.42 (—◇—), P−5Se0.84 (—△—), P−10Se1.7 (—□—), P−5VB (--▲--), P−10VB (--■--), P−10VBSe1.7 (--□--), and control (—×—). Point, mean; bar, s.d.

**Table II** Summary of side effects[a] of each regimen on human yolk sac tumour xenografted into nude mice

| *Regimen*[b] | *Ht*[c] | *WBC*[c] | *Mouse weight*[d] | *BUN*[e] |
|---|---|---|---|---|
| P−1.3 | 30 | 2.3±0.4 | 26 (+15) | 220 |
| P−1.3Se0.21 | 45 | 7.8±1.6 | 25 (+14) | 22 |
| P−2.5 | 28 | 2.8±0.5 | 25 (−10) | 248 |
| P−2.5Se0.42 | 51 | 7.0±0.9 | 26 (−12) | 25 |
| P−5 | 31 | 2.3±0.5 | 17 (−29) | 215 |
| P−5Se0.84 | 60 | 6.5±0.8 | 20 (−20) | 26 |
| P−10 | dead[f] | − | − | − |
| P−10Se1.7 | 51 | 6.0±1.6 | 20 (−20) | 28 |
| P−5VB | 25 | NT[g] | 17 (−26) | 239 |
| P−10VB | dead | − | − | − |
| P−10VBSe1.7 | 29 | NT | 19 (−27) | 35 |
| control | 40 | 11.2±3 | 32 (+35) | 21 |

[a]Toxicologic studies were performed at 22 days after the initiation of the treatment; [b]Doses in each regimen are described in **Table I** and **Materials and methods**; [c]Hematocrit (Ht) and white blood cell counts (WBC) are expressed as the mean % and the mean±s.d. × $10^{-3}\,mm^3$ of each group of mice, respectively, at the 3rd week after the initiation of treatment; [d]Changes in body weight (g) were measured weekly and expressed as the mean weight at termination of treatment (and mean % changes in each group of mice calculated from the formula; body weight after treatment/body weight before treatment × 100); [e]BUN ($mg\,dl^{-1}$) was measured with urea-GLDH method and is expressed as the mean of each group of mice; [f]dead: death due to drug toxicity; [g]NT: not tested.

The effect of co-administration of Se on reduction of CDDP-induced adverse effects is summarized in Table II. The administration of CDDP without Se caused significant elevation of BUN (5–12-fold) noted in treated mice. The significant elevation of BUN was not observed when Se was co-administered with CDDP. Mice treated with CDDP as a single agent or in combination therapy also showed a significant ($P<0.05$) decrease of peripheral WBC and haematocrit while those receiving Se showed less toxicity. The loss of body weight in the groups of mice receiving CDDP without Se was more profound than in the groups of mice receiving CDDP with Se. Although this difference was not statistically significant, it does imply that co-therapy with Se may help to minimize the drug associated weight loss toxicity.

### Discussion

In the present study, we evaluated the antitumour activity of CDDP with or without co-administration of Se against a human yolk sac tumour growing in nude mice. Our study supports the previously published findings with mouse tumours that the co-administration of Se with CDDP treatment retains antitumour activity while decreasing host toxicity (Naganuma *et al.*, 1983, 1984; Satoh *et al.*, 1985). In our experiments we used as our experimental model a human yolk sac tumour which was transplanted into nude mice. CDDP administrated as a single agent or in combination therapy demonstrated a dose dependent antitumour activity. This activity was the same with and without additional Se.

There was, however, a marked decrease in the severe adverse chemotherapy side effects such as nephrotoxicity, myeloid suppression and body weight loss observed in the groups of mice to which Se was given. As a result of a protective effect of Se on CDDP-induced lethal toxic side effects, a 1.9-fold increase in CDDP LD50, when compared with the CDDP alone, was obtained. Improvement in therapeutic index may also be obtained because the data clearly show that co-administration of Se increases the therapeutic index of CDDP since its antitumour activity is maintained but the host toxicity is reduced. This phenomenon was readily observed with the high toxic level doses of $10 \mathrm{mg\,kg^{-1}}$ CDDP (P–10, P–10Se1.7, P–10VB and P–10VBSe1.7). Mice treated with CDDP at the dose of $10 \mathrm{mg\,kg^{-1}}$, combined with Se, revealed marked tumour regression with minimal adverse side effects. In contrast, the groups of mice receiving the same dose of CDDP without Se all died due to drug toxicity within 9 days after the initiation of treatment. Some side effects could be quantitated such as elevation of BUN, decrease of body weight, reduction of haematocrit, and decrease in WBC was rarely noted in mice which received CDDP with an adequate dose of Se. We could not accumulate any information about the potential beneficial therapeutic effect of Se to prevent nausea and severe vomiting (Bodenner *et al.*, 1986).

Little information is available on the pharmacokinetics of the interaction of CDDP with Se. It was recently reported that the toxic action of CDDP was probably caused not by the coordination structure of CDDP, which is necessary for antitumour activity, but by the compound $Pt^{2+}$ arising from the decomposition of CDDP *in vivo* (Naganuma *et al.*, 1983). It would therefore by reasonable to speculate that Se may interact with toxic $Pt^{2+}$ derived from CDDP. This could happen if formation of a metal-Se complex occurred *in vivo*. Naganuma *et al.* (1987) reported that the preadministration of bismuth, which has been used as a metallothionein inducer, was effective in decreasing CDDP toxicity while antitumour activity remained. In our preliminary studies, no significant elevation of metallothionein in kidney tissues from Se-treated mice was found (data not shown). Recently diethyldithiocarbamate has been shown to decrease the CDDP induced toxicity without any reduction of antitumour activity (Bodenner *et al.*, 1986) but the explanation is unclear. From our data it can be concluded that co-administration of Se with CDDP (CDDP: Se, molar ratio, 3.5:1) is associated with the beneficial effect of decreasing CDDP mediated toxicity with concomitant-retention of anti-tumour activity. This indirectly suggests that more effective chemotherapeutic treatment of cancer in humans with CDDP may be possible if the chemotherapy includes Se.

The authors wish to thank Dr H.T. Wepsic and Dr N. Imura for their helpful advices and criticisms. This work was supported by a Grant-in-Aid for Cancer Research from the Ministry of Education, Science and Culture, Japan.

### References

BODENNER, D.L., DEDON, P.C., KENG, P.C., KATZ, J.C. & BORCH, R.F. (1986). Selective protection against *cis*-diamminedichloroplatinum(II)-induced toxicity in kidney, gut and bone marrow by diethyldithiocarbamate. *Cancer Res.*, **46**, 2751.

BORCH, R.F. & PLEASANTS, M.E. (1979). Inhibition of *cis*-platinum nephrotoxicity by diethyldithiocarbamate rescue in a rat model. *Proc. Natl Acad. Sci USA*, **76**, 6611.

EINHORN, L.H.. & DONOHUE, H.J. (1977). Improved chemotherapy in disseminated testicular cancer. *J. Urol.*, **117**, 65.

GANTHER, H.E., GOUDIE, C., SUNDE, M.L. & 4 others (1972). Selenium: Relation to decreased toxicity of methylmercury added to diet containing tuna. *Science*, **175**, 1122.

GARNETT, M.C., EMBLETON, M.J., JACOBS, E. & BALDWIN, R.F. (1983). Preparation and properties of a drug-carrier-antibody conjugate showing selective antibody-directed cytotoxicity *in vitro*. *Int. J. Cancer*, **31**, 661.

HOUCHENS, D.P., OVERJERA, A.A. & BARKER, A.D. (1979). The therapy of human tumors in athymic (nude) mice. In *Proc. symposium on the use of athymic (nude) mice in cancer research*, Houchens and Overjera (eds) p. 267, Fisher Press: New York.

JACOBS, A.J., HARRIS, M., DEPPE, C., DASGUPTA, I. & COHEN, C.J. (1982). Treatment of recurrent and persistent germ cell tumors with *cis*-platin, vinblastine and bleomycin. *Obstet. Gynecol.*, **59**, 129.

KURMAN, R.J. & NORRIS, H.J. (1976). Endodermal sinus tumor of the ovary, a clinical and pathologic analysis of 71 cases. *Cancer*, **38**, 2404.

MERRIN, C.E. (1979). Treatment of genitourinary tumors with *cis*-dichlorodiammineplatinum(II): Experience in 250 patients. *Cancer Treat. Rep.*, **63**, 1579.

NAGANUMA, A., SATOH, M. & IMURA, N. (1984). Effect of selenite on renal toxicity and antitumor activity of *cis*-diamminedichloroplatinum in mice inoculated with Ehrlich ascites tumor cell. *J. Pharm. Dyn.*, **7**, 217.

NAGANUMA, A., SATOH, M. & IMURA, N. (1987). Prevention of lethal and renal toxicity of *cis*-diamminedichloroplatinum(II) by induction of metallothionein synthesis without compromising its antitumor activity in mice. *Cancer Res.*, **47**, 983.

NAGANUMA, A., SATOH, M., YOKOYAMA, M. & IMURA, N. (1983). Selenium efficiently depressed toxic side effect of *cis*-diamminedichloroplatinum. *Res. Commun. Chem. Phatol. Pharmacol.*, **42**, 127.

NISHI, S. & HIRAI, H. (1973). Radioimmunoassay of α-fetoprotein in hepatoma, other liver diseases and pregnancy. *Gann Monogr.*, **14**, Hirai & Miyaji (eds) p. 79.

OHKAWA, K., HIBI, N. & TSUKADA, Y. (1986*a*). Evaluation of a conjugate of purified antibodies against human AFP-dextran-daunorubicin to human AFP-producing yolk sac tumor cell lines. *Cancer Immunol. Immunother.*, **22**, 81.

OHKAWA, K., TSUKADA, Y., HIBI, N., UMEMOTO, N. & HARA, T. (1986*b*). Selective *in vitro* and *in vivo* growth inhibition against human yolk sac tumor cell lines by purified antibody against human α-fetoprotein conjugated with mitomycin C via human serum albumin. *Cancer Immunol. Immunother.*, **23**, 81.

OZOLS, R.F., CORDEN, B.J., JACOB, J., WESLEY, M.N., OSTCHEGA, Y. & YOUNG, R.C. (1984). High-dose *cis*-platin in hypertonic saline. *Ann. Int. Med.*, **100**, 19.

PRESTAYKO, A.W., D'AOUST, J.C., ISSELL, B.F. & CROOKE, S.K. (1979). *Cis*-platin (*cis*-diamminedichloroplatinum II). *Cancer Treat. Rep.*, **6**, 17.

SATOH, M., NAGANUMA, A. & IMURA, N. (1985). Coadministration of selenite allows repeated injection of CDDP of high dose which completely depresses transplanted tumor in mice. In *Proc. Japanese cancer association*, 44th Annual Meeting Tokyo, p. 301.

SCULLY, R.E. (1979). Atlas of tumor pathology, 2nd series, Fas. 16. Tumors of the ovary and maldeveloped gonads. AFIP, Washington DC, p. 226.

TSUKADA, Y., BISHOF, W.K.D., HIBI, N., HIRAI, HURWITZ, E. & SELA, M. (1982*a*). Effect of a conjugate of daunomycin and antibodies to rat α-fetoprotein on the growth of α-fetoprotein-producing tumor models. *Proc. Natl Acad. Sci. USA*, **79**, 621.

TSUKADA, Y., HURWITZ, E., KASHI, R. & 4 others (1982*b*). Chemotherapy by i.v. administration of conjugates of daunomycin with monoclonal and conventional anti-rat-α-fetoprotein antibodies. *Proc. Natl Acad. Sci. USA*, **79**, 7896.

TSUKADA, Y., KATO, Y., UMEMOTO, N., TAKEDA, Y., HARA, T. & HIRAI, H. (1984). An anti-α-fetoprotein antibody-daunorubicin conjugate with a novel poly-L-glutamic acid derivative as intermediate drug carrier. *J. Natl Cancer Inst.*, **73**, 721.

TSUKADA, Y., OHKAWA, K. & HIBI, N. (1985). Suppression of human α-fetoprotein-producing hepatocellular carcinoma growth in nude mice by anti α-fetoprotein antibody-daunorubicin conjugate with a poly-L-glutamic acid derivative as intermediate drug carrier. *Br. J. Cancer*, **52**, 111.

WILLIAMS, D.S., STABLEIN, D.M., EINHORN, L.H. & 9 others (1987). Immediate adjuvant chemotherapy versus observation with treatment at relapse in pathological stage II testicular cancer. *N. Engl. J. Med.*, **317**, 1433.

WILTSHAW, E., EVANS, B.D. & HARLAND, S. (1985). Phase III randomised trial of *cis*-platin versus JM8 (carboplatin) in 112 ovarian cancer patients, stage III and IV. *Proc. Am. Soc. Clin. Oncol.*, **4**, 121 (*abstract*).